# Low back pain: what determines functional outcome at six months? An observational study

**DOI:** 10.1186/1471-2474-11-236

**Published:** 2010-10-13

**Authors:** Michele C Harms, Charles E Peers, Derek Chase

**Affiliations:** 1 Faculty of Health and Social Care Sciences, Kingston University, St Georges University of London, Cranmer Terrace, London SW17 0RE, UK; 2 NHS Plymouth, Stoke Surgery, Belmont Villas Stoke Plymouth PL3 4DP, UK; 3 GP principal, King's College Health Centre, The Strand, London WC2R 2LS, UK

## Abstract

**Background:**

The rise in disability due to back pain has been exponential with escalating medical and societal costs. The relative contribution of individual prognostic indicators to the pattern of recovery remains unclear. The objective of this study was to determine the prognostic value of demographic, psychosocial, employment and clinical factors on outcome in patients with low back pain

**Methods:**

A prospective cohort study with six-month follow-up was undertaken at a multidisciplinary back pain clinic in central London employing physiotherapists, osteopaths, clinical psychologists and physicians, receiving referrals from 123 general practitioners. Over a twelve-month period, 593 consecutive patients referred from general practice with simple low back pain were recruited. A baseline questionnaire was developed to elicit information on potential prognostic variables. The primary outcome measures were change in 24-item Roland Morris disability questionnaire score at six months as a measure of low back related functional disability and the physical functioning scale of the SF-36, adjusted for baseline scores.

**Results:**

Roland Morris scores improved by 3.8 index points (95% confidence interval 3.23 to 4.32) at six months and SF-36 physical functioning score by 10.7 points (95% confidence interval 8.36 to 12.95). Ten factors were linked to outcome yet in a multiple regression model only two remained predictive. Those with episodic rather than continuous pain were more likely to have recovered at six months (odds ratio 2.64 confidence interval 1.25 to 5.60), while those that classified themselves as non-white were less likely to have recovered (0.41 confidence interval 0.18 to 0.96).

**Conclusions:**

Analysis controlling for confounding variables, demonstrated that participants showed greater improvement if their episodes of pain during the previous year were short-lived while those with Middle Eastern, North African and Chinese ethnicity demonstrated minimal improvement. The study did not support previous findings that a wide range of factors could predict outcome.

## Background

Despite expansion of services, there are indications that the prevalence of back related disability is much higher than that reported 40 years ago [1]. Large scale surveys of workers in 31 countries, including 27 Member States of the European Union [2] have shown that 25% of workers across a range of occupations suffer from back pain. The Health and Safety Executive report a 12 month prevalence of 47% in computer users [3], and up to 12% of the population were found to consult their GP or practice nurse for back pain at least once in the year ending 31 March 2009 [4]

The direct health care costs of back pain have been estimated to be $90,600 million in the United States and £1,632 million in the UK with the largest proportion of direct medical costs spent on physical therapy [5]. The economic burden is not limited to health care but has implications for the individual and society in general with production losses and informal care reported to be more than ten times the direct cost [5]. A small subset of the population, resistant to rehabilitation and with a poor prognosis for recovery, are disproportionately heavy users of health resources.

The identification of prognostic indicators is a pre-requisite to improving the targeting of services, particularly in light of controversial results of recent studies on treatment efficacy [6-9]. Demographic factors have been consistently linked to outcome [10-13] as have psychological factors [14-16], psychosocial factors [11,12,17], clinical history [13,18] and work factors [10,12,19]. However, many studies are reported to be methodologically weak, often failing to recruit a relevant or sufficient cohort, often with less than 200 participants [20]. The large range of prognostic indicators presented in previous studies also results from the failure to consider simultaneously the major domains or other prognostic variables with which those identified might be correlated [21]. This study based in primary care addresses these issues using a multiple regression model.

Our objective was therefore to determine whether individual variables or domains were linked to recovery at six months when all identified prognostic variables were taken into account in a large population of unselected back pain patients

## Methods

### The Clinic

The lack of direct access physiotherapy for low back pain prompted the establishment by the Central London Multifund and the Westminster Primary Care Trust of a multidisciplinary community based back pain clinic. The service was to provide a complex package of care, based on published guidelines [22] largely consistent with the NICE guidelines published in May 2009 [23]. The clinic employed physiotherapists, osteopaths, clinical psychologists and patients had access to physicians, providing a treatment package that could be tailored to the needs of the individual. The commissioning included a parallel service evaluation to examine the contribution of demographic, psychosocial, clinical and work factors to the change in functioning of patients referred to the clinic.

### Participants

All 687 consecutive patients with simple low back pain or nerve root pain referred to the clinic by local general practitioners during a twelve-month period to August 2000 were eligible for inclusion in the study. Patients were excluded in line with red flag symptoms for possible serious spinal pathology. These included cauda equina symptoms, sphincter disturbance or saddle anaesthesia, non-mechanical pain, thoracic pain, a history of weight loss, widespread neurological deficit or structural deformity [22]. Patients were excluded at the point of diagnostic triage by their General Practitioner, but a further two patients with organic disease (spinal abscess and kidney disease) were excluded following attendance at the clinic.

A minimal dataset was collected for 48 patients who failed to attend their first appointment. A further 46 failed to complete the baseline questionnaire. 593 consecutive patients completed a self-administered baseline questionnaire, 55 (9%) with the assistance of an interpreter. All participants gave written informed consent and the baseline questionnaire took 30 minutes to complete. Ethical approval was granted by St Mary's Hospital Regional Ethics Committee. The research carried out was in compliance with the Declaration of Helsinki.

### Baseline patient questionnaire

Factors identified in one or more published reports as predicting functional outcome can be considered to fall into four domains: demographic factors, psychosocial factors, work characteristics and clinical history.

The selection of instruments to be included in the questionnaire (Additional file 1) was based on several considerations. Firstly, the areas identified in previous studies to have prognostic value. Secondly, the questionnaires with validity and reliability and with established use in these areas. Thirdly, a set of instruments which covered the main aspects of each domain, which complemented each other, without significant redundancy or overlap. The choice of instruments was informed by an international group of back pain researchers who recommended a standard battery of outcome measures to represent the multiple dimensions of outcome in the field of back pain [24]. The domains described included pain symptoms, back related function, generic well-being and disability. These authors anticipated that the instruments would evolve with time and that the core instrument would be sufficiently brief to allow investigators to add other measures to the battery dependent on their research interest. In this study, this core data set was expanded to provide greater breadth and depth which included adding measures of somatisation and depression.

The study followed defined criteria for methodological quality for studies of prognosis, which included participants, selected as consecutive cases, with at least one prognostic outcome available from at least 80% of study population at three month follow up or later, and with appropriate statistical adjustment [20,21].

#### Demographic factors

The baseline questionnaire included questions on age, sex, self reported height and weight, smoking history [24] and information on usual levels of physical activity before the onset of the current episode. Participants were asked how frequently (three times a week or more, once or twice a week, one to three times a month, never or hardly ever) they took part in sports or activities which were mildly energetic (eg walking, woodwork, weeding, hoeing, bicycle repair, playing darts, general housework), moderately energetic (eg scrubbing, polishing car, chopping, dancing, golf, cycling, decorating, lawn mowing, leisurely swimming) or vigorous (eg: running, hard swimming, tennis, squash, digging, cycle racing). The responses were then coded to reflect both intensity of activity and frequency of participation, an approach used in the Whitehall II study which looked at the causes of back pain in 10,308 participants [25]. A similar approach and level of coding has been recommended in a recent proposal for core outcome measures in back pain [14]. The categories used in the Office for National Statistics 1991 census classification system were used to define ethnicity.

#### Employment characteristics

The core elements relating to work as a risk factor for back pain were determined. Participants in paid employment (n = 217) were additionally asked about control over various aspects of their work content and environment using a 21-point scale [25,26]. Although some studies have reported low job satisfaction as a risk factor for sickness absence due to low back pain (27), previous work in this area [26,28,29], including that of members of the steering committee, has suggested that control over the work environment (level of decision making about own workload/flexibility/work colleagues/speed of work/environment) was an important prognostic indicator with greater importance than job satisfaction and other commonly defined measures. However, It was also recognised that physical characteristics of the task were significant, including postural and mechanical demands, so questions on the key physical elements including length of time sitting and standing, typical lifting demands and the frequency of lifting tasks were and included within the questionnaire.

#### Clinical history and presentation

A series of questions sought to characterise back pain history, including time since first onset, length of current and usual episode and the frequency of episodes within the previous twelve-month period. Data on clinical presentation including neurological signs and altered sensation, impaired reflexes and pain radiation pattern were recorded by the clinician at the first appointment. The Von Korff scale [30] was used to measure the severity of pain, comprising seven questions which combine to provide a measure of pain related disability, persistence and affective distress.

#### Psychosocial and psychological factors

Housing tenure and age on leaving full time education were recorded as an index of socio-economic status [31] and information on marital and work status was also required. The core elements in the assessment of psychological risk factors for back pain were identified. Distress/depression and somatisation are reported as having an important role in the longevity of back pain (13, 16]. The modified ZUNG Depression Inventory and the Modified Somatic Perception Questionnaire (MSPQ), as a measure of somatic anxiety, make up the Distress and Risk Assessment Method (DRAM) [32]. These are commonly used outcome measures [33-35] and were included in the questionnaire. The Modified Zung Depression Inventory is a 23-item patient-completed scale measuring depressive symptoms in back pain patients. Scores range from 0 to 69 with higher scores indicating greater depression. The MSPQ consists of 13 questions; each scored 0 to 3 with a total possible range of 0 to 39 with higher scores indicating greater somatic awareness. This approach to the measurement of psychological state was also used in the UK BEAM trial [13] and additional components were covered by other instruments included in the battery of questionnaires.

### Back pain functional outcomes

The 24-item Roland Morris Disability Questionnaire [36] was pre-specified as the primary outcome. It is among the most widely used measure of back-related function [7,13,14,17] and has been proposed as part of an international instrument for standardised use [24]. To enable a more global comparison, the physical functioning scale of the SF-36 was also recorded and comprises of 10 items on activities of daily living.

### Six month postal follow-up

Follow-up questionnaires were sent to participants six months after completing the baseline questionnaire. For non-responders a reminder and second questionnaire were sent two weeks later. Persistent non-responders were invited to complete a short version of the questionnaire over the telephone. The six-month follow-up questionnaire took 10 minutes to complete.

### Statistical Analysis

Chi square, Kruskal-Wallis and Mann Whitney analyses were used for the comparison of baseline characteristics between responders and non-responders. Differences between baseline and follow-up outcome scores were analysed using repeated measures analysis of variance. Logistic regression was used to determine odds ratios of recovery, initially for every variable independently and then in a multiple regression model.

Recovery was defined as a Roland Morris disability score of zero at six-month follow-up. An odds ratio of greater than 1 indicated that recovery was more likely to occur.

Because baseline score may be a determinant of the amount of change, the individual variables were adjusted for initial score. Inclusion of adjusted and unadjusted data is reported to avoid the biases discussed by Altman [21]. Categorical, independent variables were entered directly into the model. Data over the full range of each scale was collected, however for the purposes of the regression analyses dummy coding was used to dichotomise the independent continuous variables by their median values. This has the advantage of allowing a clear interpretation of the odds ratios and avoids the restrictive assumptions of straight-line linearity between variables. Treatment of missing data for the MSPQ and Zung indices used mean imputation where at least half the items were present. All analyses were conducted in SPSS (SPSS Inc, Chicago, Illinois) and overseen by an independent statistician.

## Results

At six months, four hundred and eighty four participants completed a follow-up questionnaire, a response rate of 82%. There were 112 persistent non-responders, despite a strict follow-up protocol of postal reminders and phone calls.

Respondents and non-respondents were similar with respect to all baseline variables with the exception that non-respondents were more likely to be male, living either in a private rental or rent-free accommodation. Participants attended an average of 6 (SD 3.7) treatment sessions.

### Primary Outcomes

**Figure 1:**
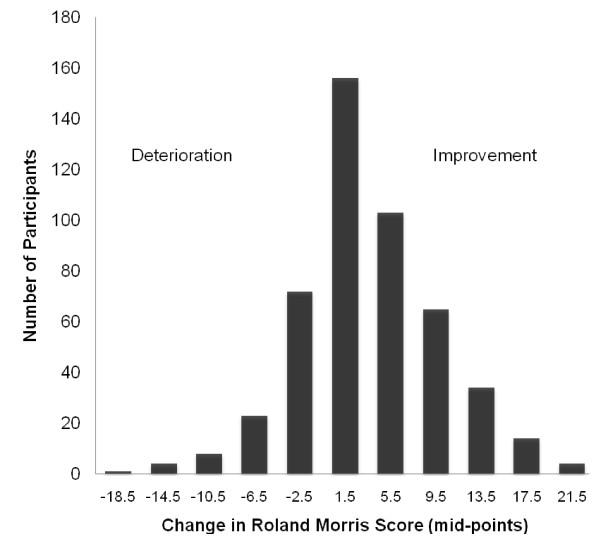
**Change in Roland Morris disability questionnaire score from baseline to six-month follow-up**.

The mean Roland Morris disability score improved by 3.8 index points (95% confidence interval 3.23 to 4.32, p < 0.001) over the six month follow-up, from 11.6 at baseline assessment to 7.8 index points at six months. The distribution of change scores is illustrated in figure 1. The SF- 36 physical functioning scale improved by 10.7 scale points (95% confidence interval 8.4 to 13.0, p < 0.001) from 49.2 at baseline assessment to 59.8 points at six months.

### Demographic factors

Persistence of symptoms at six months was predominantly associated with ethnic grouping. Participants who categorised themselves as non-white had a reduced odds ratio for recovery of 0.39 (0.20 to 0.74, p = 0.004). Those recording ethnic group as North African or Middle Eastern showed a change of less than one index point on the Roland Morris questionnaire and 2.7 points on the SF-36. There was some evidence to suggest that participants who recorded a higher frequency of exercise participation were more likely to have recovered at six month than those who rarely undertook exercise (Table 1).

**Table 1:** Demographic factors

Variable	N (%)	Missing (%)	n "0" at follow up (%)	Unadjusted odds ratio & CI	Unadjusted p-Value	Baseline Roland Morris adjusted Odds Ratio & CI	Adjusted p-value
**Age^#^**							
≥ 43 years	233 (49.4)		34 (14.6)	1.00		1.00	
< 43 years	230 (48.7)	9 (1.9)	29 (12.6)	0.84 (0.50 to 1.44)	0.517	0.61 (0.34 to 1.07)	0.61

**Sex**							
Female	274 (58.1)		31 (11.3)	1.00		1.00	
Male	197 (41.7)	1 (0.2)	34 (17.3)	1.64 (0.97 to 2.77)	0.067	1.60 (0.93 to 2.77)	0.09

**Ethnicity**							
White	294 (62.3)		51 (17.3)	1.00		1.00	
Non-white	173 (36.6)	5 (1.1)	13 (7.5)	0.39 (0.20 to 0.74)	0.004	0.52 (0.27 to 1.01)	0.06

**BMI #**							
≥ 25	212 (44.9)		27 (12.7)	1.00		1.00	
< 25	208 (44.1)	52 (11)	33 (15.9)	1.29 (0.75 to 2.24)	0.297	0.95 (0.54 to 1.70)	0.92

**Smoker**							
Never smoked	234 (49.6)		26 (11.1)	1.00		1.00	
Current or Ex-smoker	235 (49.8)	3 (0.6)	38 (16.2)	1.54 (0.90 to 2.64)	0.112	1.74 (0.99 to 3.03)	0.05

**Physical Activity**							
Low	274 (58.1)		24 (8.8)	1.00		1.00	
Medium	121 (25.6)		23 (19.0)	2.45 (1.32 to 4.53)		1.79 (0.94 to 3.40)	
High	54 (11.4)	23 (4.9)	13 (24.1)	3.30 (1.56 to 7.00)	< 0.001	1.78 (0.79 to 3.98)	0.04
							

Odds ratios of recovery (defined as a follow-up Roland Morris score = 0) for all potential predictors in the data set: (i) unadjusted (ii) adjusted for Roland Morris baseline score. Participants presenting with baseline score of 0 excluded from analysis. (N = 472)

Variables denoted with "#" have been dichotomised from a continuous scale in relation to the relevant median.

Ideally, where individual prognostic variables are found to be predictive of outcome, efficient clinical cut-off scores could be used to make decision rules about the need for treatment. This has theoretical and clinical relevance for binary variables like gender or previous surgery. However, in the case of BMI the main analysis used a grouping of data above and below the median. To give greater clinical relevance a further analysis was undertaken. The BMI is usually graded as underweight, optimal, overweight, obese and morbidly obese, rather than dichotomised as required for the main analysis. The mean change scores (with 95% confidence intervals) in the RMDQ for these categories were as follows: BMI less than 25 (underweight/optimal) 4.3 (3.4 to 5.2); BMI 25.01 to 30 (overweight) 4.0 (2.7 to 5.3); and BMI 30 and over (obese/morbidly obese) 4.3 (2.4 to 6.3). A correlation between the continuous variable BMI against the change score in the Roland Morris yielded a non-significant Pearson Product Moment coefficient of 0.05 supporting the results of the main analysis. The extreme categories (BMI less than 18.5 and more than 40.0) had too few participants to provide meaningful independent analysis. Prognostic indicators in these groups may warrant further study.

### Employment characteristics

Although we found some evidence to suggest that those in paid employment and the self employed had a greater chance of recovery (odds ratio 2.07, 1.21 to 3.54, p = 0.008), factors including control over work, the work environment and the physical characteristics of the tasks involved were not linked to recovery (Table 2). The questions addressing the physical characteristics of work were condensed to two dimensions. The question on time spent sitting was the converse of time spent walking so it was logical to reduce this to one variable. Similarly, there were few participants who recorded lifting 50 kg and those that did, also lifted 25 kg, so this was also reduced to one variable. The data provides an indication of the nature of work undertaken whether largely sedentary or involving heavy lifting.

**Table 2:** Employment characteristics

Variable	N (%)	Missing (%)	n "0" at follow up (%)	Unadjusted odds ratio & CI	Unadjusted p-Value	Baseline Roland Morris adjusted Odds Ratio & CI	Adjusted p-value
**Control over work^#^** **(0:low -21:high)**							
≥ 14	112 (51.4)		23 (20.5)	1.00		1.00	
< 14	104 (47.7)	1 (0.5)	17 (16.3)	0.76 (0.38 - 1.51)	0.496	0.86 (0.42 to 1.73)	0.82

**Sitting**							
**Some/little/none of time**	113 (51.8)		19 (35.8)	1.00		1.00	
**all or most of the time**	105 (50.1)	2 (0.9)	21 (39.0)	1.24 (0.62 - 2.46)	0.833	1.06 (0.52 to 2.15)	0.82

**Lifting 25lbs**							
**Some/little/none of time**	186 (85.3)		38 (20.4)	1.00		1.00	
**all or most of the time**	34 (15.6)	0 (0)	2 (5.9)	0.24 (0.56 - 1.06)	0.175	0.28 (0.06 to 1.25)	0.26

Odds ratios of recovery (defined as a follow-up Roland Morris score = 0) for all potential predictors in the data set: (i) unadjusted (ii) adjusted for Roland Morris baseline score. Participants presenting with baseline score of 0 excluded from analysis. (N = 217)

Variables denoted with "#" have been dichotomised from a continuous scale in relation to the relevant median.

Of the 45 participants who were in paid employment and reported being absent from work as a direct result of their back pain, only 3 (6%) reported that they were still absent at six month follow-up.

### Clinical history and Presentation

The odds ratio for recovery increased in participants who had experienced less than twelve short episodes in the past twelve months compared to those who described the nature of their episodes as continuous. Participants categorised as Grade IV on the Von Korff pain scale, indicating high levels of disability and severely limiting pain, had a reduced chance of recovery (odds ratio 0.07, 0.02 to 0.23, p < 0.001) compared to those classified as Grade I; low disability and low intensity (Table 3).

**Table 3:** Clinical history and Presentation

Variable	N (%)	Missing (%)	n "0" at follow up (%)	Unadjusted odds ratio & CI	Unadjusted p-Value	Baseline Roland Morris adjusted Odds Ratio & CI	Adjusted p-value
**Time since first episode^#^**							
Six years and over	247 (52.3)		29 (11.7)	1.00		1.00	
Up to 6 years ago	216 (45.8)	9 (1.9)	34 (15.7)	1.40 (0.82 - 2.39)	0.354	1.59 (0.91 to 2.77)	0.36

**Duration of current episode#**							
3 months or less	239 (50.6)		41 (17.2)	1.00		1.00	
Over 3 months	182 (38.6)	10 (2.1)	19 (10.4)	0.56 (0.56 - 1.01)	0.083	0.56 (0.31 to 1.02)	0.15

**Episodes in past 12 months**							
Continuous, on/off	307 (65)		28 (9.1)	1.00		1.00	
Episodic	162 (34.3)	3 (0.6)	37 (22.8)	2.95 (1.73 - 5.03)	< 0.001	2.67 (1.53 to 4.64)	0.00

**Length of usual episode**							
**12 weeks or less**	298 (63.1)		49 (16.4)	1.00		1.00	
More than 12 weeks	165 (35)	9 (1.9)	16 (9.7)	0.55 (0.30 - 0.99)	0.071	0.64 (0.35 to 1.19)	0.48

**Days of pain in last month#**							
28 days or more	234 (49.6)		24 (10.3)	1.00		1.00	
Less than 28 days	227 (48.1)	11 (2.3)	40 (17.6)	1.87 (1.09 - 3.22)	0.044	1.27 (0.71 to 2.25)	0.59

**Radiating leg pain**							
Absent	175 (37.1)		22 (12.6)	1.00		1.00	
Above knee	122 (25.8)		22 (18.0)	1.53 (0.81 - 2.91)		2.16 (1.09 to 4.30)	
Below knee	158 (33.5)	17 (3.6)	18 (11.4)	0.89 (0.46 - 1.74)	0.245	1.62 (0.79 to 3.35)	0.09

**Neurological deficit**							
No	397 (84.1)		57 (14.4)	1.00		1.00	
Yes	34 (7.2)	41 (8.7)	4 (11.8)	0.80 (0.27 - 2.34)	0.678	1.57 (0.50 to 4.94)	0.44

**Impaired reflexes**							
No	370 (78.4)		49 (13.2)	1.00		1.00	
Yes	52 (11)	50 (10.6)	6 (11.5)	0.85 (0.35 - 2.11)	0.733	1.13 (0.44 to 2.87)	0.80

**Previous treatment**							
No	172 (36.4)		29 (16.9)	1.00		1.00	
Yes	294 (62.3)	6 (1.3)	34 (11.6)	0.65 (0.38 - 1.10)	0.108	0.67 (0.38 to 1.16)	0.15

**Previous back surgery**							
No	457 (96.8)		64 (14.0)	1.00		1.00	
Yes	7 (1.5)	8 (1.7)	0 (0)	0.01 (0.0 - 2.8E+9)	0.697	0.01 (0.00 - 2.2E+9)	0.73

**Von Korff Pain Scale** Grade:							
**0 ***pain free*	0 (0)						
**I ***low disability/low intensity*	59 (12.5)		20 (33.9)	1.00		1.00	
**II ***low disability/high intensity*	157 (33.3)		19 (12.1)	0.27 (0.13 - 0.55)		0.35 (0.16 to 0.73)	
**III ***high disability-moderately limiting*	128 (27.1)		21 (16.4)	0.38 (0.19 - 0.78)		0.77 (0.35 to 1.71)	
**IV ***high disability/severely limiting*	110 (23.3)	18 (3.8)	4 (3.6)	0.07 (0.02 - 0.23)	< 0.001	0.23 (0.07 to 0.79)	0.15

**SF-36 Physical Functioning**							
≥ 50	247 (52.3)		51 (20.6)	1.00		1.00	
< 50	219 (46.4)	6 (1.3)	12 (5.5)	0.22 (0.12 - 0.43)	< 0.001	0.53 (0.24 to 1.18)	0.16

**Baseline Roland Morris^#^**							
≥ 11	256 (54.2)		15 (5.9)	1.00			
< 11	216 (45.8)	0 (0)	50 (23.1)	4.84 (2.63 - 8.90)	< 0.001		

Odds ratios of recovery (defined as a follow-up Roland Morris score = 0) for all potential predictors in the data set: (i) unadjusted (ii) adjusted for Roland Morris baseline score. Participants presenting with baseline score of 0 excluded from analysis. (N = 472)

Variables denoted with "#" have been dichotomised from a continuous scale in relation to the relevant median.

Since completing treatment, 69% of participants reported experiencing a further spell of back pain, although only 21% felt it severe enough to see either their GP or other health practitioner. These included physiotherapists, osteopaths, chiropractors, acupuncturists, orthopaedic surgeons or rheumatologists.

### Psychosocial and psychological factors

Low scores on the Zung depression inventory (odds ratio 3.43, 1.90 to 6.19, p < 0.001) and the index of somatic anxiety (odds ratio 2.36, 1.36 to 4.09, p < 0.001) were found to be linked to improvement in Roland Morris disability and SF-36 physical functioning scores, with sizable effects on both scales. However, once the individual variables were adjusted for baseline Roland Morris scores, their effect was reduced (Table 4).

**Table 4:** Psychosocial and psychological factors

Variable	N (%)	Missing (%)	n "0" at follow up (%)	Unadjusted odds ratio & CI	Unadjusted p-Value	Baseline Roland Morris adjusted Odds Ratio & CI	Adjusted p-value
**Age at leaving full time education**							
18 years or under	262 (55.5)		30 (11.5)	1.00		1.00	
Over 18 years	201 (42.6)	9 (1.9)	34 (16.9)	1.57 (0.93 - 2.67)	0.093	1.24 (0.71 to 2.15)	0.46

**Marital status**							
Married/living with partner	242 (51.3)		27 (11.2)	1.00		1.00	
Single/Divorced/ Separated/Widowed	227 (48.1)	3 (0.6)	38 (16.7)	1.60 (0.94 - 2.72)	0.082	1.46 (0.84 to 2.54)	0.18

**Housing tenure**							
Non-owner	337 (71.4)		46 (13.6)	1.00		1.00	
Owner	133 (28.2)	2 (0.4)	18 (13.5)	0.99 (0.55 - 1.78)	0.974	0.76 (0.41 to 1.39)	0.37

**Zung depression score^#^**							
≥ 23	240 (50.9)		17 (7.1)	1.00		1.00	
< 23	222 (47.0)	10 (2.1)	46 (20.7)	3.43 (1.90 - 6.19)	< 0.001	1.92 (1.01 to 3.63)	0.18

**Modified Somatic Perception score^#^**							
≥ 8	243 (51.5)		22 (9.1)	1.00		1.00	
< 8	221 (46.8)	8 (1.7)	42 (19.0)	2.36 (1.36 - 4.09)	< 0.001	1.24 (0.68 to 2.29)	0.11

**In paid employment**							
No	254 (53.8)		25 (9.8)	1.00		1.00	
Yes	217 (46.0)	1 (0.2)	40 (18.4)	2.07 (1.21 - 3.54)	0.008	1.29 (0.73 to 2.29)	0.38

Odds ratios of recovery (defined as a follow-up Roland Morris score = 0) for all potential predictors in the data set: (i) unadjusted (ii) adjusted for Roland Morris baseline score. Participants presenting with baseline score of 0 excluded from analysis. (N = 472)

Variables denoted with "#" have been dichotomised from a continuous scale in relation to the relevant median.

The scores for the MSPQ and the Zung Depression Inventory (Table 4) are comparable with other studies of similar cohorts; (Mean MSPQ: 5.6 [37], 9.7 [38], 6.7 [39]); (Mean Modified Zung Depression Index: 24.9 [37], 29.7 [38], 23.7 [39]). However, there are many ways of analysing and reporting data for psychological problems. Using the decision rules for the Distress and Risk Assessment method (DRAM) defined by Main [32] and used in the UK Beam trial [13] and other studies [33,40], patients can be classified into clusters depending on their scores on the MSPQ and the Zung Depression Inventory (Table 5).

**Table 5:** DRAM classification of participants who responded at 6 months and provided both Zung and MSPQ scores (N = 471)

Type	Decision rules	Description	N (%)	Mean (95% CI) change in RMDQ
**Normal (N)**	Modified Zung < 17	No evidence of distress or abnormal illness behaviour	151 (32)	3.2 (2.3 to 4.1)

**At Risk (R)**	Modified Zung 17 to 33 and MSPQ < 12	Slightly higher scores than normal patients, largest difference in depressive symptomatology	165 (35)	3.8 (2.8 to 4.7)

**Distressed-depressive (DD)**	Modified Zung > 33	Clear elevation on all variables, particularly high scores on depressive symptomatology	95 (20)	4.4 (3.0 to 5.8)

**Distressed-Somatic (DS)**	Modified Zung 17 to 33 and MSPQ ≥ 12	Elevation on all variables, particularly high scores on somatic symptomatology	60 (13)	3.3 (1.7 to 4.9)

The psychological profile of participants in this study, categorised using the DRAM, is comparable to similar cohorts of people with back pain (N 37%, R 42%, DD 13% and DS 9% [33]; N 24%, R 42%, DD 24% and DS 10% [40]). This suggests that the greatest proportion of participants were in the normal or at risk categories rather than in the distressed (somatic or depressive) categories.

### Multiple Regression Model

Because baseline score is a determinant of the magnitude of change, and there is likely to be co-dependency in the data, those factors found to be predictive of outcome, defined as variables with an unadjusted p-value of less than 0.1, were entered into a multiple regression (binary logistic) analysis, controlling for all other variables in the model (Table 6). Adjustment was also made for age and sex.

**Table 6:** Multiple regression analysis of predictive variables

Variable	Adjusted odds ratio and 95% confidence interval	Adjusted p-value
**Age^#^**		
≥ 43	1.00	
< 43	0.48 (0.23 to 1.02)	0.404

**Sex**		
Female	1.00	
Male	1.62 (0.83 to 3.20)	0.161

**Ethnicity**		
White	1.00	
Non-white	0.41 (0.18 to 0.96)	0.039

**Marital Status**		
Married/living with partner	1.00	
Single/divorced/separated/widowed	1.93 (0.98 to 3.79)	0.056

**Physical Activity**		
Low	1.00	
Medium	1.76 (0.80 to 3.85)	
High	1.43 (0.55 to 3.84)	0.258

**Age at leaving full time education^#^**		
18 years or under	1.00	
Over 18	1.54 (0.77 to 3.09)	0.120

**Zung depression^#^**		
≥ 23	1.00	
< 23	1.97 (0.80 to 4.83)	0.975

**Modified Somatic Perception score^#^**		
≥ 8	1.00	
< 8	0.75 (0.33 to 1.70)	0.876

**In paid employment**		
No	1.00	
Yes	0.98 (0.45 to 2.17)	0.967

**Duration of current episode**		
3 months or less	1.00	
Over 3 months	0.55 (0.24 to 1.28)	0.438

**Episodes over past 12 months**		
Continuous/on-off	1.00	
Episodic	2.64 (1.25 to 5.60)	0.005

**Length of usual episode**		
12 weeks or less	1.00	
Over 12 weeks	1.03 (0.43 to 2.50)	0.587

**Days of pain in last month**		
28 days or more	1.00	
Less than 28 days	1.02 (0.45 to 2.33)	0.730

**Von Korff Pain Scale** Grade:		
**0 ***pain free*	-	
**I ***low disability/low intensity*	1.00	
**II ***low disability/high intensity*	0.33 (0.131 to 0.86)	
**III ***high disability/-moderately limiting*	0.97 (0.36 to 2.60)	
**IV ***high disability/severely limiting*	0.19 (0.04 to 0.92)	0.354

**SF-36 Physical Functioning^#^**		
≥ 50	1.00	
< 50	0.54 (0.20 to 1.41)	0.301

**Baseline Roland Morris^#^**		
≥ 11	1.00	
< 11	1.95 (0.78 to 4.85)	0.249

Odds Ratios with 95% confidence interval for recovery including variables with unadjusted p-values of less than 0.1 in the univariate analysis, adjusted for all variables in table.

Variables denoted with "#" have been dichotomised from a continuous scale in relation to the relevant median.

Adjusted odds ratios associated with a reduced chance of recovery were linked to self-classification as 'non-white' as opposed to 'white' (0.41, 0.18 to 0.96, p = 0.039). The pattern of back pain over the previous twelve months had an impact on recovery, increasing in those who reported episodic rather than continuous pain (2.64, 1.25 to 5.60, p = 0.005) with greatest improvement in those with fewer, brief episodes of back pain. Change in Roland Morris disability scores for each sub-classification of the two variables with predictive value in the multiple regression model is shown in Table 7.

**Table 7:** Mean change in Roland Morris for ethnic classification and episodic history (n = 472)

	N (%)	Adjusted mean change in Roland Morris Disability Score	95% confidence interval	p-value
Ethnicity*				0.003
Black African/Caribbean/other	43 (9)	3.6	1.9 to 5.2	
Indian/Pakistani/Bangladeshi	34 (7)	4.3	2.5 to 6.1	
White	285 (60)	4.4	3.8 to 5.0	
Chinese	8 (2)	1.4	-2.3 to 5.2	
North African	14 (3)	2.0	-0.8 to 4.8	
Middle Eastern	52 (11)	0.9	-0.6 to 2.4	
Other	19 (4)	3.2	0.8 to 5.6	
**Missing**	17 (4)			

**Episodes in past 12 months^#^**				< 0.001
1	43 (9)	7.1	5.4 to 8.7	
2-6	87 (18)	5.1	4.0 to 6.3	
7-12	24 (5)	4.5	2.3 to 6.6	
Continuous	301 (64)	2.8	2.2 to 3.4	
Missing	17 (4)			

*Adjusted for age, sex and episodic history

^#^Adjusted for age, sex and ethnic group

## Discussion

In this large study of prognostic indicators for recovery only ethnic grouping and periodicity of the participant's back pain were linked to recovery at six months. The results do not support the value of commonly identified determinants of outcome within demographic, psychosocial, employment and clinical domains. The results illustrate the importance of controlling confounding variables and the adjusted analysis provides an estimate of the independent effect of each variable, providing a measure of whether it contains additional prognostic information [21].

To our knowledge, this is one of the largest studies of prognostic indicators for recovery from back pain, with the highest completion to follow-up, investigating the relative contribution of predictive factors from domains often viewed in isolation.

Although data was generated from a single centre, comparison with data from other primary care studies suggests that the participants in our study are representative of the patients with back pain in primary care [7,41]. The measurement of the impact of cultural differences on change scores is limited by the relative size of the ethnic groups who demonstrated little benefit, predominantly those who classified themselves as being from North African, Chinese or Middle Eastern countries. Although interpreters accompanied many participants, it is possible that language barriers and cultural differences in the experience and report of pain may have had some influence. The intermittent nature of back pain may also have had a bearing on the results and would depend on the number of participants experiencing an episode at the point of follow-up. The treatment package offered to all patients at the clinic comprised of the same basic components and the clinician completed a record of each treatment session. The pragmatic nature of this observational study meant that individual treatments varied to some degree but are comparable to the treatment options specified in the recently published NICE guidelines on the management of persistent, non-specific low back pain [23].

Whilst work injury and compensation status have been thought to influence the course of back pain, a recent systematic review found insufficient evidence to establish the importance of compensation on aspects of recovery [11]. Only a small proportion of the sample in this study were in paid employment and off work as a result of their back pain and it was therefore not considered one of the core predictors for this sample. However, this may need to be considered in a demographically different sample.

The Fear-Avoidance Beliefs Questionnaire [42] was not selected as a core instrument, although many of the items of this 16-item questionnaire were similar to those covered. 'Despite the prevalent focus on fear' a recent systematic review found little evidence to link fear-avoidance with poor prognosis, however the authors did report a growing consensus that distress/depression plays an important role [16]. We were mindful that the length of the questionnaire, which was already substantial, could become prohibitive. However, future studies may benefit from including these aspects in greater depth in their battery of questionnaires. It is appreciated that there may be gaps in data collected, although these are not anticipated to be substantial [15].

The improvement in Roland Morris (3.8 index points) and SF-36 scales (10.7 points) suggests significant clinical recovery. The Medical Research Council funded UK BEAM trial specified a 2.5 [43] point change on the Roland Morris disability score as a clinically significant change, far smaller than the mean change seen in this study. The results differ from previously reported research which found links between demographic factors including age, sex and height, or pattern of activity [10-13] and from those linking outcome to psychological [14] or psychosocial factors [11,17]. Chronic pain-related disability results in learned behaviours which can become apparent within the first few weeks of onset. Whether psychosocial changes only become apparent as a history of back pain develops has yet to be demonstrated.

Back pain history has been recognised as a strong predictor of future episodes [18]. However, in the adjusted model, the only variable linked to recovery was the continuous or intermittent nature of the participant's pain. In this study, no evidence was found to suggest that recovery was affected by physical exposure or by the degree of control experienced within the working environment, contrasting with previously cited indicators [10,19,44], but in agreement with one systematic review [45]. Future research should test this predictive model on a new dataset to determine its prognostic strength,

## Conclusions

The results suggest that it is possible to identify patients at presentation who are high risk for persistent disabling symptoms and those who are likely to recover, information essential to the successful targeting of services. It is important to determine whether those patients shown to have a reduced likelihood of recovery should be targeted for more intensive intervention or managed by alternative methods, whilst valuable resources may be better employed on others with a greater chance of recovery. Although an analysis of changes in Roland Morris disability scores suggest that a number of prognostic variables are linked to outcome, once a model is used which adjusts for the confounding effects of all significant variables, including treatment variables, only two contained additional, independent prognostic information. Participants improved more if their episodes of pain during the previous year were short-lived while those with Middle Eastern and Chinese ethnicity demonstrated minimal improvement. The reasons for this require further investigation. In this report, both adjusted and unadjusted data are reported for clarity, but it is also important to remember that the baseline Roland Morris score itself may be a reasonable determinant of outcome at six months. The study did not support previous evidence that a wide range of factors could predict outcome.

## Competing interests

DC was *GP principal and chairman *of the Central London Multifund and a member of the steering group. The Multifund part funded this work. Otherwise the researchers were independent and there are no competing interests.

## Authors' contributions

MH was jointly responsible for conception and design of the study, co-ordination, data analysis and interpretation, drafting and revising manuscript. CP acted in an advisory capacity for clinical implementation and clinical data acquisition. DC was chairman of the commissioning agency with joint responsibility for conception, design and interpretation of results. In addition to the functions above, the authors formed part of the steering group with a collective responsibility to oversee the conduct of the study. All authors read and approved the final manuscript.

## Pre-publication history

The pre-publication history for this paper can be accessed here:

http://www.biomedcentral.com/1471-2474/11/236/prepub

## Supplementary Material

Additional file 1 **The Marylebone Back Pain Clinic Questionnaire**. Baseline Questionnaire. Click here for file

## References

[B1] Harkness EF Macfarlane GJ Silman AJ McBeth J Is musculoskeletal pain more common now than 40 years ago?: two population-based cross-sectional studies Rheumatology 2005 44 890 895 10.1093/rheumatology/keh599 15784630

[B2] Parent-Thirion A Macías EF Hurley J Vermeylen G European Foundation for the Improvement of Living and Working Conditions. 4th European Working Conditions Survey 2007 Luxembourg: Office for Official Publications of the European Communities

[B3] Melrose AS Graveling RA Cowie H Ritchie P Hutchison P Mulholland RM Better Display Screen Equipment (DSE) work-related ill health data 2007 Institute of Occupational Medicine, Health and Safety Executive

[B4] General Practice - Practice Team Information: Back pain. Information Services, NHS National Services Scotland 2009 http://isdscotland.org/isd/3703.html

[B5] Dagenais S Caro J Haldeman S A systematic review of low back pain cost of illness studies in the United States and internationally Spine J 2008 8 8 20 10.1016/j.spinee.2007.10.005 18164449

[B6] Frost H Lamb SE Doll HA Carver PT Stuart-Brown S Randomised controlled trial of physiotherapy compared with advice for low back pain BMJ 2004 329 708 11 10.1136/bmj.38216.868808.7C 15377573 PMC518892

[B7] Lamb S Hansen Z Lall R Castelnuovo E Withers E Nicols V Potter R Underwood M Group cognitive behavioural treatment for low-back pain in primary care: a randomised controlled trial and cost-effectiveness analysis Lancet 2010 375 916 923 10.1016/S0140-6736(09)62164-4 20189241

[B8] Hansen Z Daykin A Lamb SE A cognitive-behavioural programme for the management of low back pain in primary care: a description and justification of the intervention used in the Back Skills Training Trial (BeST; ISRCTN 54717854) Physiotherapy 2010 96 87 94 10.1016/j.physio.2009.09.008 20420955

[B9] Jackson A Hettinga DM Mead J Mercer C Using consensus methods in developing clinical guidelines for exercise in managing persistent low back pain Physiotherapy 2009 95 302 311 10.1016/j.physio.2009.08.001 19892095

[B10] Poiraudeau S Rannou F Le Henanff A Coudeyre E Rozenberg S Huas D Martineau C Jolivet-Landreau I Revel M Ravaud P Outcome of subacute low back pain: influence of patients' and rheumatologists' characteristics Rheumatology 2006 45 718 723 10.1093/rheumatology/kei231 16377729

[B11] Iles RA Davidson M Taylor NF Psychosocial predictors of failure to return to work in non-chronic non-specific low back pain: a systematic review Occup Environ Med 2008 65 507 517 10.1136/oem.2007.036046 18417552

[B12] Leclerc A Gourmelen J Chastang JF Plouvier S Niedhammer I Lanoë JL Level of education and back pain in France: the role of demographic, lifestyle and physical work factors Int Arch Occup Environ Health 2008 82 643 652 10.1007/s00420-008-0375-4 18956210 PMC2793406

[B13] Underwood MR Morton V Farrin A Do baseline characteristics predict response to treatment for low back pain? Secondary analysis of the UK BEAM dataset [ISRCTN32683578] Rheumatology 2007 46 1297 1302 10.1093/rheumatology/kem113 17522096

[B14] Pincus T Santos R Breen A Burton AK Underwood MR For the Multinational musculoskeletal inception cohort study collaboration A Review and Proposal for a Core Set of Factors for Prospective Cohorts in Low Back Pain: A Consensus Statement Arthritis Rheum 2008 59 14 24 10.1002/art.23251 18163411

[B15] Foster N Thomas E Bishop A Dunn KM Main CJ Distinctiveness of psychological obstacles to recovery in low back pain patients in primary care Pain 2010 148 398 406 10.1016/j.pain.2009.11.002 20022697 PMC2831173

[B16] Pincus T Vogel S Burton AK Santos R Field AP Fear avoidance and prognosis in back pain: A systematic review and synthesis of current evidence Arthritis Rheum 2006 54 3999 4010 10.1002/art.22273 17133530

[B17] Keeley P Psychosocial predictors of health related quality of life and GP consultation in people with CLBP J Psychosom Res 2004 56 612 10.1016/j.jpsychores.2004.04.168

[B18] Neubauer E Junge A Pirron P Seemann H Schiltenwolf M HKF-R 10 - Screening for predicting chronicity in acute low back pain (LBP): A prospective clinical trial Eur J Pain 2006 10 559 566 10.1016/j.ejpain.2005.08.002 16202634

[B19] Ghaffari M Alipour A Farshad AA Jensen J Josephson M Vingard E Effect of psychosocial factors on low back pain in industrial workers Occup Med 2008 58 341 347 10.1093/occmed/kqn006 18296687

[B20] Pengel LHM Herbert RD Maher CG Refshauge KM Acute low back pain: systematic review of its prognosis BMJ 2003 327 323 325 10.1136/bmj.327.7410.323 12907487 PMC169642

[B21] Altman DG Systematic reviews of evaluations of prognostic variables BMJ 2001 323 224 8 10.1136/bmj.323.7306.224 11473921 PMC1120839

[B22] Rosen M Report of a Clinical Standards Advisory Group Committee on Back Pain 1994 HMSO London

[B23] Savigny P Kuntze S Watson P Underwood M Ritchie G Cotterell M Hill D Browne N Buchanan E Coffey P Dixon P Drummond C Flanagan M Greenough C Griffiths M Halliday-Bell J Hettinga D Vogel S Walsh D Low Back Pain: Early management of persistent non-specific low back pain 2009 London: National Collaborating Centre for Primary Care and Royal College of General Practitioners. NICE Guidelines http://www.nice.org.uk/nicemedia/pdf/CG88fullguideline.pdf accessed 15.4.2010

[B24] Deyo RA Battie M Beurskens AJHM Bombardier C Croft P Koes B Malmivaara A Roland M Von Korff M Waddell G Outcome measures for low back pain research: A proposal for standardized use Spine 1998 23 2003 2013 10.1097/00007632-199809150-00018 9779535

[B25] Stress and Health Study. Department of Epidemiology and Public Health http://www.ucl.ac.uk/whitehallII/pdf/Questionnaire_S3.pdf

[B26] Hemingway H Shipley MJ Stansfeld S Marmot M Sickness absence from back pain, psychosocial work characteristics and employment grade among office workers Scand J Work Environ Health 1997 23 121 129 9167235 10.5271/sjweh.189

[B27] Hoogendoorn WE Bongers PM de Vet HCW Ariëns GAM van Mechelen W High physical work load and low job satisfaction increase the risk of sickness absence due to low back pain: results of a prospective cohort study Occup Environ Med 2002 59 323 328 10.1136/oem.59.5.323 11983847 PMC1740286

[B28] North FM Syme LS Feeney A Shipley M Marmot M Psychosocial Work Environment and Sickness Absence among British Civil Servants: The Whitehall II Study Am J Public Health 1996 86 332 340 10.2105/AJPH.86.3.332 8604757 PMC1380513

[B29] Bosma H Marmot MG Hemingway H Nicholson AG Brunner E Stansfeld A Low job control and risk of coronary heart disease in Whitehall II (prospective cohort) study BMJ 1997 314 558 65 9055714 10.1136/bmj.314.7080.558 PMC2126031

[B30] Von Korff M Ormel J Keefe F Dworkin SF Grading the severity of chronic pain Pain 1992 50 133 149 10.1016/0304-3959(92)90154-4 1408309

[B31] Dalstra JAA Kunst AE Mackenbach JP The EU Working Group on Socioeconomic Inequalities in Health A comparative appraisal of the relationship of education, income and housing tenure with less than good health among the elderly in Europe Soc Sci Med 2006 62 2046 2060 10.1016/j.socscimed.2005.09.001 16221515

[B32] Main CJ Wood PLR Hollis S Spanswick CC Waddell G The distress and risk assessment method: A simple patient classification to identify distress and evaluate the risk of poor outcome Spine 1992 17 42 52 10.1097/00007632-199201000-00007 1531554

[B33] Daubs M Patel A Willick S Richard K Hansen P Petron D Brodke DS Clinical Instinct vs. Standardized Questionnaire: The Spine Specialists Ability to Detect Psychological Distress Spine J 2008 8 Suppl 1 1S 10.1016/j.spinee.2008.06.00218942170

[B34] Melloh M Aebli N Elfering A Röder C Zweig T Barz T Herbison P Hendrick P Bajracharya S Stout K Theis J Development of a screening tool predicting the transition from acute to chronic low back pain for patients in a GP setting: Protocol of a multinational prospective cohort study BMC Musculoskeletal Disorders 2008 9 167 10.1186/1471-2474-9-167 19099569 PMC2630319

[B35] Fairbank JCT Frost H Wilson MacDonald J Yu LM Barker KL Collins R The MRC Spine Stabilisation Trial: A randomised controlled trial to compare surgical stabilisation of the lumbar spine versus an intensive rehabilitation programme on outcome in patients with chronic low back pain BMJ 2005 330 1233 1239 10.1136/bmj.38441.620417.8F 15911537 PMC558090

[B36] Roland M Morris R A study of the natural history of back pain. Part I: Development of a reliable and sensitive measure of disability in low-back pain Spine 1983 8 141 144 10.1097/00007632-198303000-00004 6222486

[B37] Hay EM Mullis R Lewis M Vohora K Main CJ Watson P Dziedzic KV Sim J Minns Lowe C Croft PR Comparison of physical treatments versus a brief pain-management programme for back pain in primary care: a randomised clinical trial in physiotherapy practice Lancet 2005 365 2024 2030 10.1016/S0140-6736(05)66696-2 15950716

[B38] Laslett M Oberg B Aprill CN McDonald B Zygapophysial joint blocks in chronic low back pain: A test of Revel's model as a screening test BMC Musculoskeletal Disorders 2004 5 43 10.1186/1471-2474-5-43 15546487 PMC534802

[B39] Carragee EJ Psychological and functional profiles in select subjects with low back pain Spine J 2001 1 198 204 10.1016/S1529-9430(01)00050-X 14588348

[B40] Hope P Forshaw MJ Assessment of Psychological Distress Is Important in Patients Presenting with Low Back Pain Physiotherapy 1999 85 563 570 10.1016/S0031-9406(05)61250-3

[B41] Klaber Moffett J Jackson DA Gardiner ED Randomized trial of two physiotherapy interventions for primary care neck and back pain patients: 'McKenzie' *vs *brief physiotherapy pain management Rheumatology 2006 45 1514 1521 10.1093/rheumatology/kel339 17062645

[B42] Chou R Shekelle P Will this patient develop persistent disabling low back pain? JAMA 2010 303 1295 302 10.1001/jama.2010.344 20371789

[B43] UK BEAM Trial Team United Kingdom back pain exercise and manipulation (UK BEAM) randomised trial: effectiveness of physical treatments for back pain in primary care BMJ 2004 329 1377 1384 10.1136/bmj.38282.669225.AE 15556955 PMC535454

[B44] Karjalainen K Malmivaara A Mutanen P Pohjolainen T Roine R Hurri H Outcome Determinants of Subacute Low Back Pain Spine 2003 28 2634 2640 10.1097/01.BRS.0000099097.61495.2E 14652481

[B45] Hartvigsen J Ling S Leboeuf-Yde C Bakketeig L Psychosocial factors at work in relation to low back pain and consequences of low back pain; a systematic, critical review of prospective cohort studies Occup Environ Med 2004 61 1 e2 14691283 PMC1757801

