# Supplementary material for: Low back pain: what determines functional outcome at six months? An observational study
Source: BMC Musculoskelet Disord. 2010 Oct 13;11:236. doi: 10.1186/1471-2474-11-236 (PMC2973928; doi:10.1186/1471-2474-11-236)
Supplement: Additional file 1 — The Marylebone Back Pain Clinic Questionnaire. Baseline Questionnaire. [file 1471-2474-11-236-S1.DOC]

# THE BACK PAIN STUDY


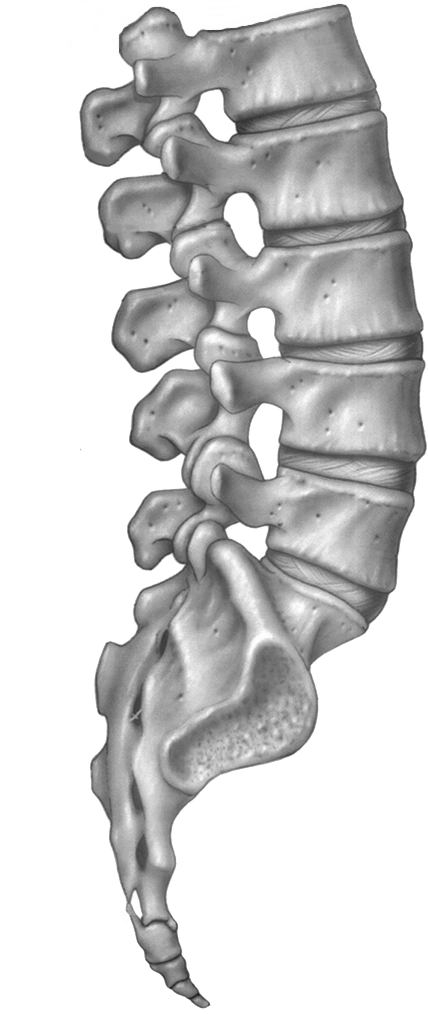


THE MARYLEBONE BACK PAIN CLINIC

# PARKSIDE HEALTH TRUST

#

CENTRAL LONDON MULTIFUND

# MARYLEBONE PRIMARY CARE GROUP

# KENSINGTON & CHELSEA AND WESTMINSTER

# HEALTH AUTHORITY

**CONFIDENTIAL**

The Marylebone Low Back Pain Clinic has been set up so that you can be seen quickly. In the past, you may have waited over six months to be seen in a hospital department. We want to find out whether your back pain improves when you are treated at the clinic

We are interested in how your back pain affects you and your life. Your views are very important and will be used to improve the service we offer. Your progress will be monitored very closely and you will be given the most up-to-date forms of treatment.

If you are happy, in six months time we will send you a second questionnaire in the post. It is important that you fill this in and return it to us in the envelope provided. If we do not hear from you, we will telephone you and may send you another copy of the questionnaire.

You do not have to take part in this study if you do not want to. If you decide not to take part you may withdraw at any time without having to give a reason. Your decision whether to take part or not will not affect your care and management in any way. The data collected during the trial is confidential and individual records will only be seen by the investigator.

| Are you happy for us to send you a questionnaire in six months time? | Yes ❑ |
| --- | --- |
|  | No ❑ |

Name ______________________________

Signature ______________________________

Date ______/______/_____

This questionnaire should only take about 30 minutes to complete and can be given to the researcher before you go for treatment

**Thank you for completing this questionnaire**

**General Information**

| How old were you when you had your first spell of back pain | _______ yrs |
| --- | --- |

|  | 1 episode | 2 – 6 episodes | 7 – 12 episodes | Continuous (on and off) over the year |
| --- | --- | --- | --- | --- |
| How many spells of back pain have you had over the **last 12 months?** | ❑ | ❑ | ❑ | ❑ |

|  | Less than 1 day | 1-7 days | 1-4 weeks | 5-8 weeks | 9-12 weeks | More than 12 weeks |
| --- | --- | --- | --- | --- | --- | --- |
| In general, did each spell last ? | ❑ | ❑ | ❑ | ❑ | ❑ | ❑ |

| How many days of back pain have you had over the **last month**? | _______ days of pain |
| --- | --- |

|  | Less than 1 week | 1-4 weeks | 5-8 weeks | 9-12 weeks |
| --- | --- | --- | --- | --- |
| How long in total did you have back pain over the **last 3 months**? | ❑ | ❑ | ❑ | ❑ |

|  | Yes | No |
| --- | --- | --- |
| 1. Have you had any previous treatment for back pain? | ❑ | ❑ |
| 1. Have you had any operations on your back? | ❑ | ❑ |

| Your Height | ______ ft ______ins |
| --- | --- |
| Your Weight | ______st ______lbs |

| Are you currently: | Married or living with a partner | ❑ |
| --- | --- | --- |
|  | Single | ❑ |
|  | Divorced or Separated | ❑ |
|  | Widowed | ❑ |

| To which of the following ethnic groups do you consider that you belong? | | | |
| --- | --- | --- | --- |
| Black-Caribbean | ❑ | Black-African | ❑ |
| Black-other | ❑ | Indian | ❑ |
| Pakistani | ❑ | Bangladeshi | ❑ |
| White | ❑ | Chinese | ❑ |
| Other | ❑ | Please specify ________________ | |

| This question relates to where you are living now. Do you (and/or your partner): | | |
| --- | --- | --- |
|  | Own your present home | ❑ |
|  | Rent it from the local council | ❑ |
|  | Rent it from a housing association | ❑ |
|  | Rent it privately or rent free | ❑ |
|  | None of the above | ❑ |

| 1. Please list any pills, medicines or tonics that you have taken in the last 14 days | |
| --- | --- |
| Name of medication | What is it for? |
| **__________________________** | **__________________________** |
| **__________________________** | **__________________________** |
| **__________________________** | **__________________________** |
| **__________________________** | **__________________________** |
| **__________________________** | **__________________________** |

| Smoking History *For our study, a regular smoker is someone who has smoked at least one cigarette a day for at least one year* | | | |
| --- | --- | --- | --- |
|  | Have you ever been a regular smoker of Cigarettes or Cigars? | Yes❑ | No ❑ |
|  | If yes, what age did you start smoking? | ________yrs | |
|  | How many cigarettes do you or did you smoke a day? | ________cigarettes | |
|  | Do you smoke now? | Yes❑ | No ❑ |
|  | If no, at what age did you stop smoking? | ________yrs | |

| 1. How would you rate your back pain on a 0-10 scale at the present time, that is **right now**, where 0 is “no pain” and 10 is ‘pain as bad as could be’? | | | | | | | | | | | | | | |
| --- | --- | --- | --- | --- | --- | --- | --- | --- | --- | --- | --- | --- | --- | --- |
|  | No pain | | | | | | |  | |  | | | Pain as bad could be | |
|  | 0 | 1 | 2 | 3 | 4 | 5 | 6 | | 7 | | 8 | 9 | | 10 |

| 1. In the past 6 months, how intense was your **worst pain** rated on a 0-10 scale where 0 is “no pain” and 10 is ‘pain as bad as could be’? | | | | | | | | | | | | | | |
| --- | --- | --- | --- | --- | --- | --- | --- | --- | --- | --- | --- | --- | --- | --- |
|  | No pain | | | | | | |  | |  | | | Pain as bad could be | |
|  | 0 | 1 | 2 | 3 | 4 | 5 | 6 | | 7 | | 8 | 9 | | 10 |

| 1. In the past 6 months, **on average**, how intense was your pain rated on a 0-10 scale where 0 is “no pain” and 10 is ‘pain as bad as could be’? (That is, your usual pain at times you were experiencing pain)? | | | | | | | | | | | | | | |
| --- | --- | --- | --- | --- | --- | --- | --- | --- | --- | --- | --- | --- | --- | --- |
|  | No pain | | | | | | |  | |  | | | Pain as bad could be | |
|  | 0 | 1 | 2 | 3 | 4 | 5 | 6 | | 7 | | 8 | 9 | | 10 |

| 1. About how many days in the last 6 months have you been kept from your usual activities (work, school or housework) because of back pain? | | | | | | | | | | |
| --- | --- | --- | --- | --- | --- | --- | --- | --- | --- | --- |
|  | …………………………days | | | | | | | | | |
|  |  |  |  |  |  |  |  |  |  |  |

| 1. In the past 6 months, how much has back pain **interfered with your daily activities** rated on a 0-10 scale where 0 is “no interference” and 10 is ‘unable to carry on any activities’? | | | | | | | | | | | | | | |
| --- | --- | --- | --- | --- | --- | --- | --- | --- | --- | --- | --- | --- | --- | --- |
|  | No interference | | | | | | |  | |  | | | Unable to carry on any activities | |
|  | 0 | 1 | 2 | 3 | 4 | 5 | 6 | | 7 | | 8 | 9 | | 10 |

| 1. In the past 6 months, how much has back pain **changed your ability** to take part in recreational, social and family activities where 0 is “no change” and 10 is ‘extreme change’? | | | | | | | | | | | | | | |
| --- | --- | --- | --- | --- | --- | --- | --- | --- | --- | --- | --- | --- | --- | --- |
|  | No change | | | | | | |  | |  | | | Extreme change | |
|  | 0 | 1 | 2 | 3 | 4 | 5 | 6 | | 7 | | 8 | 9 | | 10 |

| 1. In the past 6 months, how much has back pain **changed your ability to work** (including housework) where 0 is “no change” and 10 is ‘extreme change’? | | | | | | | | | | | | | | |
| --- | --- | --- | --- | --- | --- | --- | --- | --- | --- | --- | --- | --- | --- | --- |
|  | No change | | | | | | |  | |  | | | Extreme change | |
|  | 0 | 1 | 2 | 3 | 4 | 5 | 6 | | 7 | | 8 | 9 | | 10 |

| 1. How old were you when you | 14 or younger | 15-16 years old | 17-18 years old | 19-20 years old | 21 or older |
| --- | --- | --- | --- | --- | --- |
| finished full time education | ❑ | ❑ | ❑ | ❑ | ❑ |

| What professional qualifications do you have? | | | |
| --- | --- | --- | --- |
| Matriculation | ❑ | GNVQ | ❑ |
| Masters/PhD | ❑ | NVQ | ❑ |
| Degree/Diploma | ❑ | CSE | ❑ |
| City & Guilds | ❑ | Highers | ❑ |
| GCSE/O Levels | ❑ | A Levels | ❑ |
| None | ❑ |  |  |

|  | | Yes | No |
| --- | --- | --- | --- |
|  | Are you in paid employment at the moment? | ❑ | ❑ |

| 1. What is (or was) your main occupation ____________________________________________ |
| --- |

| 1. If you are **not** in paid employment at present, how would you classify yourself (please Tick one only) | | |
| --- | --- | --- |
| Unemployed | ❑ |  |
| Retired | ❑ |  |
| Long-term sick | ❑ |  |
| Housewife/husband | ❑ |  |
| Student | ❑ |  |
| Other (please specify) _____________________________________ | | |

|  | | Yes | No |
| --- | --- | --- | --- |
|  | Are you on sick leave at the moment | ❑ | ❑ |
|  | If YES, is this because of your back? | ❑ | ❑ |

| 1. If YES, when were you last well enough to work? | | | | | | |
| --- | --- | --- | --- | --- | --- | --- |
| Less than 1 week ago | 1-2  weeks ago | 2-4  weeks ago | 1-3  months ago | 3-6  months ago | 6-12 months ago | More than 1 year ago |
| ❑ | ❑ | ❑ | ❑ | ❑ | ❑ | ❑ |

|  | | | Yes | No |
| --- | --- | --- | --- | --- |
|  | Do you plan to return to your present job when you are well enough to return to work? | | ❑ | ❑ |
|  | | | to take a different job? | not to work again? |
|  | | IF NO, do you plan: | ❑ | ❑ |

| How many days during the last 12 months | None | 1-5 days | 6-30 days | 31-60 days | 61-90 days | More than 90 days |
| --- | --- | --- | --- | --- | --- | --- |
| have you been off work because of your  back? | ❑ | ❑ | ❑ | ❑ | ❑ | ❑ |
| When we send you a questionnaire in six months time, we will be asking you about how much time you have had off since coming to the clinic. It would help very much if you kept a record. | | | | | | |

|  | |  | All of the time | Most of the time | Some/a little of the time | None of the time |
| --- | --- | --- | --- | --- | --- | --- |
|  | How much sitting does your work involve? | | ❑ | ❑ | ❑ | ❑ |
|  | How much standing or walking does your work involve? | | ❑ | ❑ | ❑ | ❑ |
|  | How often do you lift 25lbs on the job (about the weight of 12 telephone directories)? | | ❑ | ❑ | ❑ | ❑ |
|  | How often do you lift 50lbs on the job about the weight of 25 telephone directories)? | | ❑ | ❑ | ❑ | ❑ |

| About your position at work – how often do the following statements apply?:  *Please answer all questions* | | | | |  | | |
| --- | --- | --- | --- | --- | --- | --- | --- |
|  | | Often | Sometimes | Seldom | | Never / Almost never | |
|  | Others take decisions concerning my work | ❑ | ❑ | ❑ | | | ❑ |
|  | I have a good deal of say in decisions about work | ❑ | ❑ | ❑ | | | ❑ |
|  | I have a say in my own work speed | ❑ | ❑ | ❑ | | | ❑ |
|  | My working time can be flexible | ❑ | ❑ | ❑ | | | ❑ |
|  | I can decide when to take a break | ❑ | ❑ | ❑ | | | ❑ |
|  | I have a say in choosing with whom I work | ❑ | ❑ | ❑ | | | ❑ |
|  | I have a great deal of say in planning my work environment | ❑ | ❑ | ❑ | | | ❑ |

| How often do you take part in sports or activities that are: *Tick one box on each line* | | | | | |
| --- | --- | --- | --- | --- | --- |
|  | | 3 times a week or more | Once or twice a week | About 1 to 3 times a month | Never / Hardly ever |
|  | **Mildly energetic**  (eg: walking, woodwork, weeding, hoeing, bicycle repair, playing darts, general housework) | ❑ | ❑ | ❑ | ❑ |
|  | Moderately energetic (eg: scrubbing, polishing car, chopping, dancing, golf, cycling, decorating, lawn mowing, leisurely swimming) | ❑ | ❑ | ❑ | ❑ |
|  | **Vigorous**  (eg: running, hard swimming, tennis, squash, digging, cycle racing) | ❑ | ❑ | ❑ | ❑ |

**About your back**

When your back hurts, you may find it difficult to do some of the things you normally do. The list contains some sentences that people have used to describe themselves when they have back pain. When you read them, you may find that some stand out because they describe you over the *last 24 hours*. As you read the list, think of yourself *today.* When you read a sentence that describes you, put a Tick against it. If the sentence does not describe you, then leave the space blank and go in to the next one. Remember, only Tick the sentence if you are sure it describes you over the *last 24 hours*.

| Tick all sentences that apply to you *today* | | |
| --- | --- | --- |
|  | I stay at home most of the time because of my back problem or leg pain | ❑ |
|  | I change position frequently to try and get my back or leg comfortable | ❑ |
|  | I walk more slowly than usual because of my back problem or leg pain | ❑ |
|  | Because of my back problem or leg pain I am not doing any of the jobs that I usually do around the house | ❑ |

|  | Because of my back problem or leg pain, I use a handrail to get upstairs | ❑ |
| --- | --- | --- |
|  | Because of my back problem or leg pain, I lie down to rest more often | ❑ |
|  | Because of my back problem or leg pain, I have to hold onto something to get out of an easy chair | ❑ |
|  | Because of my back problem or leg pain, I try to get other people to do things for me | ❑ |

|  | I get dressed more slowly than usual because of my back problem or leg pain | ❑ |
| --- | --- | --- |
|  | I only stand up for short periods of time because of my back problem or leg pain | ❑ |
|  | Because of my back problem or leg pain, I try not to bend or kneel down | ❑ |
|  | I find it difficult to get out of a chair because of my back problem or leg pain | ❑ |

|  | My back or leg is painful almost all of the time | ❑ |
| --- | --- | --- |
|  | I find it difficult to turn over in bed because of my back problem or leg pain | ❑ |
|  | My appetite is not very good because of my back problem or leg pain | ❑ |
|  | I have trouble putting on my socks (or stockings) because of the pain in my back or leg | ❑ |

|  | I only walk short distances because of my back or leg pain | ❑ |
| --- | --- | --- |
|  | I sleep less well because of my back problem or leg pain | ❑ |
|  | Because of my back or leg pain, I get dressed with help from someone else | ❑ |
|  | I sit down for most of the day because of my back problem or leg pain | ❑ |

|  | I avoid heavy jobs around the house because of my back problem or leg pain | ❑ |
| --- | --- | --- |
|  | Because of my back or leg pain, I am more irritable and bad tempered with people than usual | ❑ |
|  | Because of my back or leg pain, I go upstairs more slowly than usual | ❑ |
|  | I stay in bed most of the time because of my back or leg pain | ❑ |

|  | Because of my back pain, my sexual activity is decreased | ❑ |
| --- | --- | --- |
|  | I keep rubbing or holding areas of my body that hurt or are uncomfortable | ❑ |
|  | Because of my back, I am doing less of the daily work around the house than I would usually do | ❑ |
|  | I often express concern to other people over what might be happening to my health | ❑ |

This section concerns your overall health and daily activities

| 1. In general would you say your health is:-  *Please indicate* ***one*** *only* | | | | |
| --- | --- | --- | --- | --- |
| Excellent | Very good | Good | Fair | Poor |
| ❑ | ❑ | ❑ | ❑ | ❑ |

| 1. Compared to one year ago, how would you rate your health in general now ?   *Please indicate* ***one*** *only* | |
| --- | --- |
| Much better now than one year ago | ❑ |
| Somewhat better than one year ago | ❑ |
| About the same as one year ago | ❑ |
| Somewhat worse than one year ago | ❑ |
| Much worse now than one year ago | ❑ |

| The following questions are about activities you might do during a typical day. Does your health now limit you in these activities ? If so, how much ? *Tick one box on each line* | | | | |
| --- | --- | --- | --- | --- |
|  | | Yes, limited a lot | Yes, limited a little | No, not limited at all |
|  | Vigorous activities, such as running, lifting heavy objects, participating in strenuous sports | ❑ | ❑ | ❑ |
|  | Moderate activities such as moving a table, pushing a vacuum cleaner, bowling or golf | ❑ | ❑ | ❑ |
|  | Lifting or carrying groceries | ❑ | ❑ | ❑ |
|  | Climbing several flights of stairs | ❑ | ❑ | ❑ |
|  | Climbing one flight of stairs | ❑ | ❑ | ❑ |
|  | Bending, kneeling or stooping | ❑ | ❑ | ❑ |
|  | Walking more than one mile | ❑ | ❑ | ❑ |
|  | Walking half a mile | ❑ | ❑ | ❑ |
|  | Walking one hundred yards | ❑ | ❑ | ❑ |
|  | Bathing or dressing yourself | ❑ | ❑ | ❑ |

| During the past 4 weeks, have you had any of the following problems with your work or other regular daily activities as a result of your **physical health?** *Answer yes or no to each question* | | | | | |
| --- | --- | --- | --- | --- | --- |
|  | | Yes | | No | |
|  | Cut down the amount of time you spent on work or other activities | | ❑ | | ❑ |
|  | Accomplished less than you would like | | ❑ | | ❑ |
|  | Were limited in the kind of work or other activities | | ❑ | | ❑ |
|  | Had difficulty performing the work or other activities (for example it took extra effort) | | ❑ | | ❑ |

| During the past 4 weeks, have you had any of the following problems with your work or other regular daily activities as a result of any **emotional problems (such as feeling depressed or anxious)?** *Answer yes or no to each question* | | | |
| --- | --- | --- | --- |
|  | | Yes | No |
|  | Cut down the amount of time you spent on work or other activities | ❑ | ❑ |
|  | Accomplished less than you would like | ❑ | ❑ |
|  | Didn’t do work or other activities as carefully as usual | ❑ | ❑ |

| 1. During the past 4 weeks, to what extent has your **physical health or emotional problems** interfered with your normal social activities with family, friends, neighbours or groups ? *Tick one box* | | | | |
| --- | --- | --- | --- | --- |
| Not at all | Slightly | Moderately | Quite a bit | Extremely |
| ❑ | ❑ | ❑ | ❑ | ❑ |

| 1. How much bodily pain have you had during the past 4 weeks? *Tick one box* | | | | | |
| --- | --- | --- | --- | --- | --- |
| None | Very mild | Mild | Moderate | Severe | Very severe |
| ❑ | ❑ | ❑ | ❑ | ❑ | ❑ |

| 1. During the past 4 weeks, how much did pain interfere with your normal work (including both work outside the home and housework)? *Tick one box* | | | | |
| --- | --- | --- | --- | --- |
| Not at all | A little bit | Moderately | Quite a bit | Extremely |
| ❑ | ❑ | ❑ | ❑ | ❑ |

| How much of the time, during the past 4 weeks? *Tick one box on each line* | | | | | | | |
| --- | --- | --- | --- | --- | --- | --- | --- |
|  | | All of the time | Most of the time | A good bit of the time | Some of the time | A little of the time | None of the time |
|  | Did you feel full of life | ❑ | ❑ | ❑ | ❑ | ❑ | ❑ |
|  | Have you been a very nervous person | ❑ | ❑ | ❑ | ❑ | ❑ | ❑ |
|  | Have you felt so down in the dumps that nothing could cheer you up | ❑ | ❑ | ❑ | ❑ | ❑ | ❑ |
|  | Have you felt calm and peaceful | ❑ | ❑ | ❑ | ❑ | ❑ | ❑ |
|  | Did you have a lot of energy | ❑ | ❑ | ❑ | ❑ | ❑ | ❑ |
|  | Have you felt downhearted and low | ❑ | ❑ | ❑ | ❑ | ❑ | ❑ |
|  | Did you feel worn out | ❑ | ❑ | ❑ | ❑ | ❑ | ❑ |
|  | Have you been a happy person | ❑ | ❑ | ❑ | ❑ | ❑ | ❑ |
|  | Did you feel tired | ❑ | ❑ | ❑ | ❑ | ❑ | ❑ |

| 1. During the past 4 weeks, how much of the time has your physical health or emotional problems interfered with your **social activities** (like visiting friends, relatives etc.)? *Tick one box* | | | | |
| --- | --- | --- | --- | --- |
| All of the time | Most of the time | Some of the time | A little of the time | None of the time |
| ❑ | ❑ | ❑ | ❑ | ❑ |

| How TRUE or FALSE is each of the following statements for you: *Tick one box on each line* | | | | | | |
| --- | --- | --- | --- | --- | --- | --- |
|  | | Definitely true | Mostly true | Don’t know | Mostly false | Definitely false |
|  | I seem to get ill more easily than other people | ❑ | ❑ | ❑ | ❑ | ❑ |
|  | I am as healthy as anybody I know | ❑ | ❑ | ❑ | ❑ | ❑ |
|  | I expect my health to get worse | ❑ | ❑ | ❑ | ❑ | ❑ |
|  | My health is excellent | ❑ | ❑ | ❑ | ❑ | ❑ |

Please describe how you have felt during the PAST WEEK by ticking the appropriate box. Please answer all questions. *Do not think too long before answering*.

|  | | Not at all | A little, slightly | A great deal, quite a bit | Extremely, could not have been worse |
| --- | --- | --- | --- | --- | --- |
|  | Heart rate increase | ❑ | ❑ | ❑ | ❑ |
|  | Feeling hot all over | ❑ | ❑ | ❑ | ❑ |
|  | Sweating all over | ❑ | ❑ | ❑ | ❑ |
|  | Sweating in a particular part of the body | ❑ | ❑ | ❑ | ❑ |
|  | Pulse in neck | ❑ | ❑ | ❑ | ❑ |
|  | Pounding in head | ❑ | ❑ | ❑ | ❑ |
|  | Dizziness | ❑ | ❑ | ❑ | ❑ |
|  | Blurring of vision | ❑ | ❑ | ❑ | ❑ |
|  | Feeling faint | ❑ | ❑ | ❑ | ❑ |
|  | Everything appearing unreal | ❑ | ❑ | ❑ | ❑ |
|  | Nausea | ❑ | ❑ | ❑ | ❑ |
|  | Butterflies in stomach | ❑ | ❑ | ❑ | ❑ |
|  | Pain or ache in stomach | ❑ | ❑ | ❑ | ❑ |
|  | Stomach churning | ❑ | ❑ | ❑ | ❑ |
|  | Desire to pass water | ❑ | ❑ | ❑ | ❑ |
|  | Mouth becoming dry | ❑ | ❑ | ❑ | ❑ |
|  | Difficulty swallowing | ❑ | ❑ | ❑ | ❑ |
|  | Muscles in neck aching | ❑ | ❑ | ❑ | ❑ |
|  | Legs feeling weak | ❑ | ❑ | ❑ | ❑ |
|  | Muscles twitching or jumping | ❑ | ❑ | ❑ | ❑ |
|  | Tense feeling across forehead | ❑ | ❑ | ❑ | ❑ |
|  | Tense feeling in jaw muscles | ❑ | ❑ | ❑ | ❑ |

Please indicate for each of these questions which answer best describes how you have been feeling recently

|  | | Rarely or none of the time  (less than 1 day/ week) | Some or little of the time  (1-2 days/week) | A moderate amount of time    (3-4 days/ week) | Most of the time  (5-7 days/ week) |
| --- | --- | --- | --- | --- | --- |
|  | I feel downhearted and sad | ❑ | ❑ | ❑ | ❑ |
|  | Morning is when I feel best | ❑ | ❑ | ❑ | ❑ |
|  | I have crying spells or feel like it | ❑ | ❑ | ❑ | ❑ |
|  | I have trouble getting to sleep at night | ❑ | ❑ | ❑ | ❑ |
|  | I feel that nobody cares | ❑ | ❑ | ❑ | ❑ |
|  | I eat as much as I used to | ❑ | ❑ | ❑ | ❑ |
|  | I still enjoy sex | ❑ | ❑ | ❑ | ❑ |
|  | I notice I am losing weight | ❑ | ❑ | ❑ | ❑ |
|  | I have trouble with constipation | ❑ | ❑ | ❑ | ❑ |
|  | My heart beats faster than usual | ❑ | ❑ | ❑ | ❑ |
|  | I get tired for no reason | ❑ | ❑ | ❑ | ❑ |
|  | My mind is as clear as it used to be | ❑ | ❑ | ❑ | ❑ |
|  | I tend to wake up too early | ❑ | ❑ | ❑ | ❑ |
|  | I find it easy to do the things I used to | ❑ | ❑ | ❑ | ❑ |
|  | I am restless and cant keep still | ❑ | ❑ | ❑ | ❑ |
|  | I feel hopeful about the future | ❑ | ❑ | ❑ | ❑ |
|  | I am more irritable than usual | ❑ | ❑ | ❑ | ❑ |
|  | I find it easy to make a decision | ❑ | ❑ | ❑ | ❑ |
|  | I feel quite guilty | ❑ | ❑ | ❑ | ❑ |
|  | I feel that I am useful and needed | ❑ | ❑ | ❑ | ❑ |
|  | My life is pretty full | ❑ | ❑ | ❑ | ❑ |
|  | I feel that others would be better off if I were dead | ❑ | ❑ | ❑ | ❑ |
|  | I am still able to enjoy the things I used to | ❑ | ❑ | ❑ | ❑ |

Your own health state today

By placing a tick in each group below, please indicate which statement best describes your own health state today. Do not tick more than one box in each group.

| Mobility | I have no problems in walking about  I have some problems in walking about  I am confined to bed | | ❑  ❑  ❑ |
| --- | --- | --- | --- |
|  | | |  |
| Self-Care | I have no problems with self-care  I have some problems washing or dressing myself  I am unable to wash or dress myself | | ❑  ❑  ❑ |
|  | | |  |
| Usual Activities (eg: work, study, housework, family or leisure activities) | I have no problems with performing my usual activities  I have some problems with performing my usual activities  I am unable to perform my usual activities | | ❑  ❑  ❑ |
|  | | |  |
| Pain/Discomfort | I have no pain or discomfort  I have moderate pain or discomfort  I have extreme pain or discomfort | | ❑  ❑  ❑ |
|  | | |  |
| Anxiety/Depression | | I am not anxious or depressed  I am moderately anxious or depressed  I am extremely anxious or depressed | ❑  ❑  ❑ |

| 1. How much do you think treatment at the clinic will improve your pain on a 0-10 scale, where 0 is “will make no difference” and 10 is ‘will make me completely better’? | | | | | | | | | | | | | | |
| --- | --- | --- | --- | --- | --- | --- | --- | --- | --- | --- | --- | --- | --- | --- |
|  | Will make  no difference | | | | | | |  | |  | | | Will make me completely better | |
|  | 0 | 1 | 2 | 3 | 4 | 5 | 6 | | 7 | | 8 | 9 | | 10 |
